# The Transcription Factor NFATc1 Supports the Rejection of Heterotopic Heart Allografts

**DOI:** 10.3389/fimmu.2018.01338

**Published:** 2018-06-12

**Authors:** Johannes Baur, Christoph Otto, Ulrich Steger, Stefan Klein-Hessling, Khalid Muhammad, Tobias Pusch, Krisna Murti, Rhoda Wismer, Christoph-Thomas Germer, Ingo Klein, Nora Müller, Edgar Serfling, Andris Avots

**Affiliations:** ^1^Department of General, Visceral, Vascular, and Pediatric Surgery, University Hospital of Würzburg, Würzburg, Germany; ^2^Experimental Surgery, Department of General, Visceral, Vascular, and Pediatric Surgery, University Hospital of Würzburg, Würzburg, Germany; ^3^Department of Molecular Pathology, Institute of Pathology, Comprehensive Cancer Center Mainfranken, Julius-Maximilians University of Würzburg, Würzburg, Germany; ^4^Transplant and Hepatobiliary Surgery, Department of General, Visceral, Vascular, and Pediatric Surgery, University Hospital of Würzburg, Würzburg, Germany; ^5^Institute for Virology and Immunobiology, University of Würzburg, Würzburg, Germany

**Keywords:** NFATc1, transplantation, heterologous, CD8^+^ T cells, ChIPseq, metabolism

## Abstract

The immune suppressants cyclosporin A (CsA) and tacrolimus (FK506) are used worldwide in transplantation medicine to suppress graft rejection. Both CsA and FK506 inhibit the phosphatase calcineurin (CN) whose activity controls the immune receptor-mediated activation of lymphocytes. Downstream targets of CN in lymphocytes are the nuclear factors of activated T cells (NFATs). We show here that the activity of NFATc1, the most prominent NFAT factor in activated lymphocytes supports the acute rejection of heterotopic heart allografts. While ablation of NFATc1 in T cells prevented graft rejection, ectopic expression of inducible NFATc1/αA isoform led to rejection of heart allografts in recipient mice. Acceptance of transplanted hearts in mice bearing NFATc1-deficient T cells was accompanied by a reduction in number and cytotoxicity of graft infiltrating cells. In CD8^+^ T cells, NFATc1 controls numerous intracellular signaling pathways that lead to the metabolic switch to aerobic glycolysis and the expression of numerous lymphokines, chemokines, and their receptors, including Cxcr3 that supports the rejection of allogeneic heart transplants. These findings favors NFATc1 as a molecular target for the development of new strategies to control the cytotoxicity of T cells upon organ transplantation.

## Introduction

The worldwide application of cyclosporin A (CsA) and tacrolimus (FK506) as immune suppressants in transplantation medicine saved the lives of thousands of patients. CsA and FK506 prevent organ rejection by inhibiting the activity of phosphatase calcineurin (CN) that orchestrates the activation of peripheral lymphocytes upon antigen contact (1). In cells, CsA binds to CypA, a low molecular weight immunophilin, and these complexes bind to CN and suppress its enzymatic activity (2). However, due to the numerous side effects caused by CsA and FK506, sustained applications of both immune suppressants not only lead to severe complications in transplant patients, in particular to nephrotoxicity, but also to neurotoxicity, hypertension, fibrosis, and cancerogenesis (3, 4). Although CsA and FK506 inhibit in lymphocytes primarily the activation of nuclear factors of activated T cell (NFAT) transcription factors (TFs), NFATs are not the only TFs whose activity is affected by CsA. Since CN supports the induction of NF-κB factors upon TCR stimulation, CsA interferes also with their TCR-mediated activation (5, 6).

Due to the many deleterious side effects of CsA and FK506 in therapy, numerous searches have been undertaken for novel CN inhibitors, or for inhibitors that suppress directly the activation of NFATs. One set of such inhibitors are cell-penetrating peptides that interfere with the interaction between CN and NFATs (7). Based on the affinity-driven selection of peptides corresponding to the main interaction site of NFATs with CN (8), several peptide versions of general structure PxIxIT (where x represents any natural amino acid) were shown to inhibit the interaction between CN and NFATs (9). They interfere with the binding of CN to an NFAT peptide that is highly conserved between all NFAT proteins and, thereby, those inhibitory peptides do not distinguish between individual NFAT members. Since, however, the individual NFAT proteins differ remarkably in their activity *in vivo* (10), for therapeutic interventions inhibitors have to identified that allow the specific block of individual NFAT proteins.

Among the treatments that suppressed the rejection of allogeneic transplanted hearts in mice (3) there are several therapies that affect the activation of NFATs and/or their targets in T cells. Apart from the use of CN inhibitors CsA and FK506, metabolic inhibitors and the inactivation of NF-κB or *Cxcr3*, a chemokine receptor gene, led to acceptance of transplanted allogeneic hearts (11–13). We showed previously that NF-κB factors support the induction of short NFATc1/αA in B lymphocytes (14). We will show here that NFATc1 ablation prolongs allograft survival, impairs the metabolic switch from oxidative phosphorylation (OXPHOS) to aerobic glycolysis in activated CD8^+^ T cells and the expression of chemokine receptor *Cxcr3* in CTLs. These data indicate NFATc1 as a key factor in activated T cells that controls the rejection of transplanted allogeneic hearts.

## Materials and Methods

### Mice and Isolation of T Cells

Male C57BL/6J (B6, H-2^b^) and BALB/c (H-2^d^) wild-type (WT) mice were purchased from Janvier (France). *Nfatc1^flx/flx^* and *Nfatc2*^−/−^ mouse lines have been described previously. *Nfatc1^flx/flx^* mice were crossed with *CD4-cre* mice for inactivating the *Nfatc1* gene in all T cells, and with *mb1-cre* mice in all B cells (15–20). In *dlck-cre x Nfatc1^flx/flx^* mice, the *Nfatc1* gene is inactivated in peripheral T cells. In mice of the *dlck-cre x Nfatc1^flx/flx^x caNfatc1-STOP^flx/flx^* line, a constitutively active version of NFATc1/αA is expressed from the *Rosa26* locus upon removal of a “floxed” STOP sequence and inactivation of endogenous *Nfatc1* gene in peripheral T cells (21, 22). *Nfatc1P2Δ* mice carry an *Nfatc1 P2* promoter deletion and, due to a CMV-promoter-driven cre are deficient for P2-directed transcripts [see Figure S1 in Supplementary Material and Ref. (23)]. Bacterial artificial chromosome (BAC) transgenic (tg) mice expressing NFATc1/A-Bio protein [and BirA, the biotin-ligase from *E. coli* (24)] have been described previously in Ref. (25). All mice were maintained in the Central Animal Facility of the Medical Faculty (ZEMM), University of Wuerzburg, according to the institutional guidelines (acceptance AKZ 55.2-2531.01-80/10 from 22.10.2010).

For CD8^+^ T cell isolation, the “CD8 (Ly2) microbeads, mouse” kit (Miltenyi Biotech) was used. For αCD3/CD28 stimulation, 5 µg CD3ε (clone 145-2C11) and 2 µg CD28 (clone 37.51) (both BD Pharmingen) were used to coat multi well plates. T cells were also stimulated with 10 ng/ml tetradecanoylphorbol-13-acetate and 0.5 µM ionomycin (normally for 5 h).

### Heterotopic Murine Heart Transplantation

Abdominal heterotopic heart transplantation into mice was performed as described previously in Ref. (26).

### Isolation of Graft-Infiltrating Cells (GICs)

Heart grafts cut into small pieces were incubated in 100 U/ml collagenase at 37°C for 30 min. Cells were washed with phosphate-buffered saline (PBS), counted, and purified on a Ficoll-Hypaque gradient.

### Histologic and Immune Histochemical Analysis

Freshly explanted heart grafts were fixed in 4% paraformaldehyde and embedded in paraffin. For hematoxylin–eosin staining, 4-µm sections were de-paraffinized with xylene, rehydrated in absolute ethanol, stained in hematoxylin solution, and counter-stained with eosin. For immune histochemical staining, de-paraffinized and rehydrated sections (1 µm) were heated for antigen unmasking in 10 mM sodium citrate buffer (pH 6.0), and stained with αCXCR3 (CD183, #bs-2209R, Bioss Antibodies, Inc. MA, USA), diluted 1:200 in antibody (Ab) dilutent (DAKO, Hamburg, Germany) at 4°C overnight. Sections were washed in PBS and incubated with 1:100 diluted horseradish-labeled goat anti-rabbit IgG (DAKO, P0448) at room temperature for 1 h. Staining was developed by adding 3,3′diaminobenzidine (DAB ready to use, DAKO) and counterstaining was done with hematoxylin.

### Confocal Microscopy of CD8^+^ T Cells

Upon isolation, splenic CD8^+^ T cells were stimulated with αCD3/CD28 Abs for 24 h, attached to poly-l-Lysin-coated chamber μ-slides, fixed in 4% formaldehyde, permeabilized with 0.2% Triton-X100, and blocked with 5% BSA. Samples were incubated with primary mouse anti-NFATc1 Ab 7A6 in 1% BSA at 4° overnight.

### RNA Seq Transcriptome Analysis

Graft-infiltrating cells were isolated from heart grafts at day 5 after transplantation. Purification of RNA from GICs and transcriptome assays were performed at TRON GmbH (Mainz, Germany) as described previously in Ref. (27).

### ChIP Seq Assays

CD8^+^ T cells from BAC tg mice expressing BirA, or from tg mice expressing BirA and Nfatc1/A-Bio, a Bio-tagged version of NFATc1/αA [(25); Figure S4 in Supplementary Material] were differentiated to CTLs *in vitro*. Upon re-stimulation with T + I (5 ng/ml and 0.5 µM) for 5 h, chromatin from 1 × 10^7^ fixed cells was prepared and sonicated, followed by precipitation of chromatin on streptavidin beads (M-280, #11205D, Thermo Fisher Scientific, MA, USA) (28). After cDNA library preparation (using a NEBNext^®^ Ultra™ DNA Library Prep Kit for Illumina^®^) sequencing was performed as a 50-bp single read run on an Illumina HiSeqTM2500 (Illumina, San Diego, CA, USA) using a TruSeq^®^ Rapid SBS Kit v2 and a HiSeq Rapid Flow Cell v2. 50 bp sequence reads that passed the Illumina quality filtering were aligned to the mouse genome assembly version of July 2007 (NCB I37/mm9), using the map with Bowtie for Illumina 1.1.2. Results were visualized with the “Integrative Genomics Viewer” IGV version 2.3.81 (29).

### Immunoblotting

Western blots were performed using the NFATc1-specific mAbs 7A6 and NFATc2 mAb (# 556602 and 5062574, BD Pharmingen) for NFAT detection.

### Flow Cytometry

Lymphocyte stainings were performed by incubation for 20 min on ice using conjugated the following mAbs (eBioscience, San Diego, CA, USA): CD8-FITC (#11-0081-82), CD4-FITC(#MCD0401), CXCR3-APC (#17-1831-82), CCR5-PE (#12-1951-81), CD62L-PE (#12-0621-81), CD44-APC (#17-0441-81), CD25-APC (#17-0251-82), B220 FITC (#11-0452-82), CD5-PE (#12-0051-82), CD23-APC (#MCD2305).

### Cytotoxicity Assays

The mineral oil induced murine MOPC-315.BMP2.FUGLW55 plasmacytoma cell line (H2^b^) expressing eGFP and luciferase was used as target cell line. To measure luciferase during coculture the assays were performed by cultivating MOPC-*luciferase* target cells together with equal numbers of GICs isolated from WT B6 or *Nfatc1^flx/flx^x CD4-cre* recipient mice during the acute rejection phase (d6, effector/target ratio 4/1, followed by 20 or 36 h incubation). After centrifugation, the cell pellet was washed once with PBS and finally re-suspended in 100 µl harvesting buffer to lyse cell membranes. 50 µl of supernatant was transferred into a white, non-transparent 96-well plate. The LUMIstar Omega was primed with 1 ml of ready-to-use luciferin solution before luciferase-activity measurement was performed. 50 µl of luciferin solution per well were automatically added to the sample and measurement was performed.

### Extracellular Flux Assays

The extra cellular acidification rate (ECAR) of CD8^+^ T cells was measured in a XF96 analyzer (Seahorse Bioscience) as described previously in Ref. (25).

### Statistical Analysis

Student’s *t*-test or the Mann–Whitney *U* test was used for statistical analysis with the software GraphPad Prism 6. *P* values above 0.05 were considered not significant.

## Results

### NFATc1 Supports the Rejection of Allogeneic Heart Transplants

**Figure 1:**
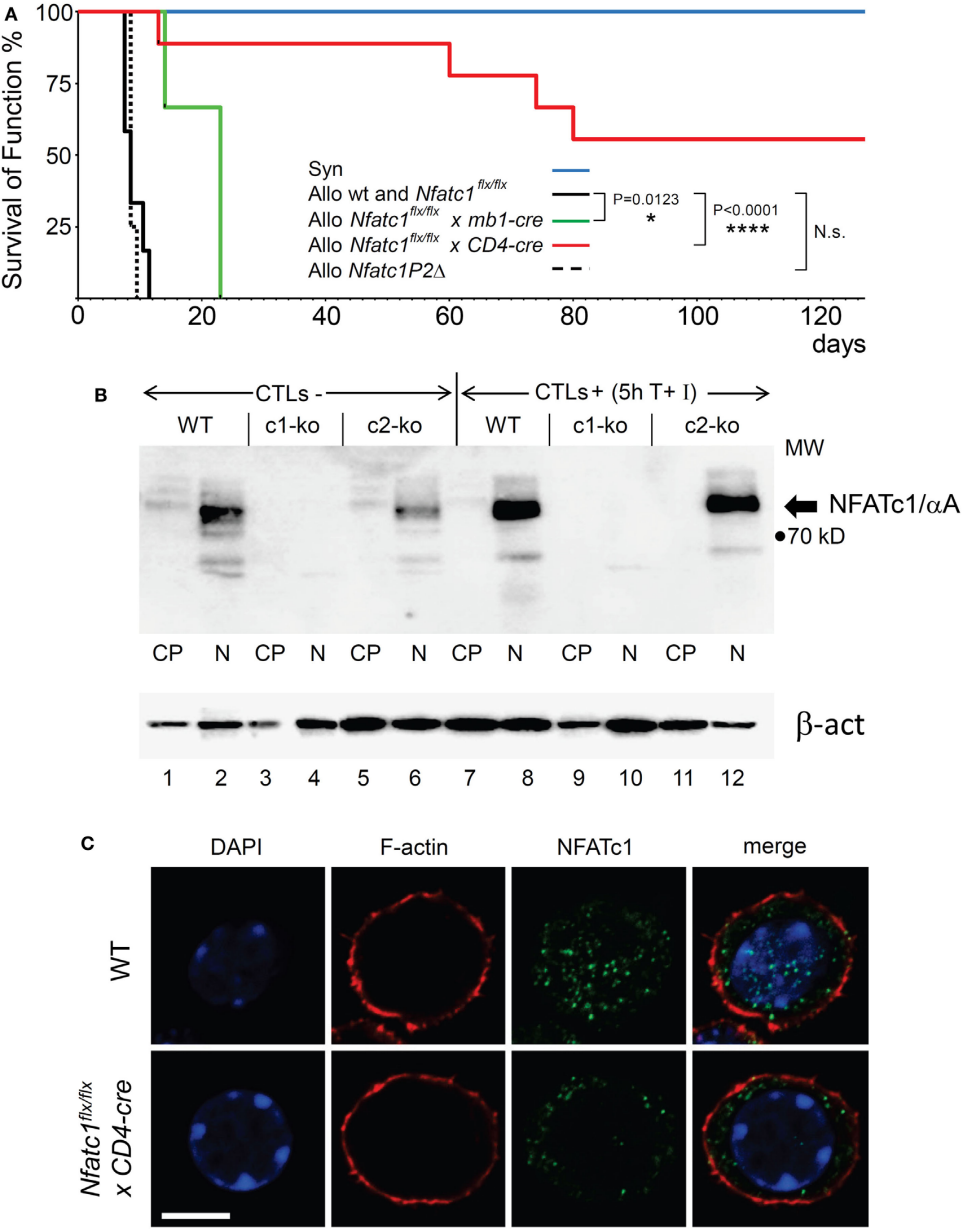
NFATc1 deficiency in T cells prevents acute rejection of allogeneic heterotopic heart transplants. **(A)** Syngeneic (C57BL/6, Syn) or fully allogenic (BALB/c, Allo) heart grafts were transplanted into wild-type (WT) C57BL6, *Nfatc1^flx/flx^ x mb1-cre, Nfatc1^flx/flx^ x CD4-cre*, or *Nfatc1P2Δ* recipient mice (*n* = 5–9 recipients per group). While *Nfatc1^flx/flx^ x mb1-cre* mice bear NFATc1-deficient B cells, *Nfatc1^flx/flx^ x CD4-cre* mice bear NFATc1-deficient CD4^+^ and CD8^+^ T cells. *Nfatc1P2Δ* mice carry an *Nfatc1 P2* promoter deletion and, therefore, are deficient for P2-directed transcripts (23). The survival of heart function was monitored daily during the first 3 weeks after transplantation, then four times per week. **(B)** Representative western blot showing the efficient NFATc1 ablation in cytotoxic T cells (CTL) from *Nfatc1^flx/flx^ x CD4-cre* (c1-ko) mice. Cytoplasmic (CP) and nuclear proteins (N) from CTLs of WT, *Nfatc1^flx/flx^ x CD4-cre* (c1-ko) and *Nfatc2*^−/−^ (c2-ko) mice were fractionated. For the generation of CTLs *in vitro*, splenic CD8^+^ T cells were stimulated by αCD3/CD28 antibodies (Abs) for 3 days, followed by culture in IL-2 containing medium for further 3 days. They were left un-induced (CTL-) or induced by T + I (CTL^+^) for 5 h (see Materials and Methods). The prominent band of NFATc1/αA, the inducible, short NFATc1 isoform, is indicated. **(C)** Staining of splenic CD8^+^ T cells from WT or *Nfatc1^flx/flx^xCD4-cre* mice activated for 24 h by αCD3/CD28 Abs *in vitro*. Cytospins of cells were stained with Abs raised against F-actin or NFATc1 (7A6 mAb), and counter-stained by DAPI. One typical stain of more than 50 cells analyzed is shown. Bar, 10 µm.

In a previous report on the survival of heterotopic heart and skin transplants, in *Nfatc2-* or *Nfatc3-*deficient recipient mice a moderate prolongation of allogeneic transplant survival was described in Ref. (30). These observations prompted us to investigate whether NFATc1, the most prominent NFAT factor in activated peripheral T and B cells, plays a role in the rejection of allogeneic organ transplants. In a model of heterotopic heart transplantation, we used *Nfatc1^flx/flx^* mice crossed with the *CD4-cre* or *mb1-cre* mouse lines to ablate the expression of NFATc1 in peripheral T or B lymphocytes, respectively (18, 20, 22). Fully allogeneic hearts from donor BALB/c mice transplanted into WT B6 recipient mice are normally rejected within 7 days by the recipient’s immune system. However, BALB/c heart allografts transplanted into *Nfatc1^flx/flx^xCD4-cre* mice bearing both NFATc1-deficient CD4^+^ and CD8^+^ T cells survived significantly longer for more than 50 days, and more than 50% of the allografts for more than 120 days. In contrast, transplantation of allogeneic hearts into *Nfatc1^flx/flx^x mb1-cre* mice bearing *Nfatc1*^−/−^ B cells had only a minor positive effect on the survival of transplanted allogeneic hearts (Figure 1A).

In lymphocytes, the *Nfatc1* gene is expressed in six individual α- and β-isoforms at the RNA and protein levels. These are generated under the control of two alternate promoters, P1 and P2, and polyA sites, pA1 and pA2, and, therefore, differ in their N- and C-terminal peptides (Figure S1 in Supplementary Material) (31). In T cells from *Nfatc1^flx/flx^x CD4-cre* mice, the expression of all isoforms should be ablated (Figure 1B and unpubl. data), but upon staining of immunoblots or cytospins prepared with NFATc1^−/−^ CD8^+^ T cells using the 7A6 NFATc1 mAb various shorter protein bands or stained dots were detected. However, these NFATc1-like proteins were not imported into the nucleus upon activation of NFATc1^−/−^ T cell cells (Figure 1C) and, therefore, should play a minor, if any role in transcriptional regulation.

To identify the role of individual NFATc1 isoforms in transplant rejection, we crossed *Nfatc1-P2^flx/flx^* mice created in our laboratory (32) with CMV-cre mice for the deletion of *P2* promoter and *exon 2* sequences. In such *Nfatc1P2Δ* mice, the generation of NFATc1/β isoforms was inhibited [see Figure S1 in Supplementary Material and Ref. (23)]. *Nfatc1P2Δ* mice were born at expected Mendelian ratios, were viable and developmentally indistinguishable from their *Nfatc1P2^flx/flx^* littermates, and their lymphoid compartments remained unaffected. Both T and B cells from *Nfatc1P2Δ* mice could be normally activated *ex vivo* and did not reveal any defects in proliferation or induction of Activation-Induced Cell Death (Figure S2 in Supplementary Material). However, the rejection of allogeneic hearts upon transplantation into *Nfatc1P2Δ* mice was detected on the same day as upon transplantation into WT B6 mice (Figure 1A). This led us to conclude that NFATc1/β proteins play a minor role in graft rejection.

**Figure 2:**
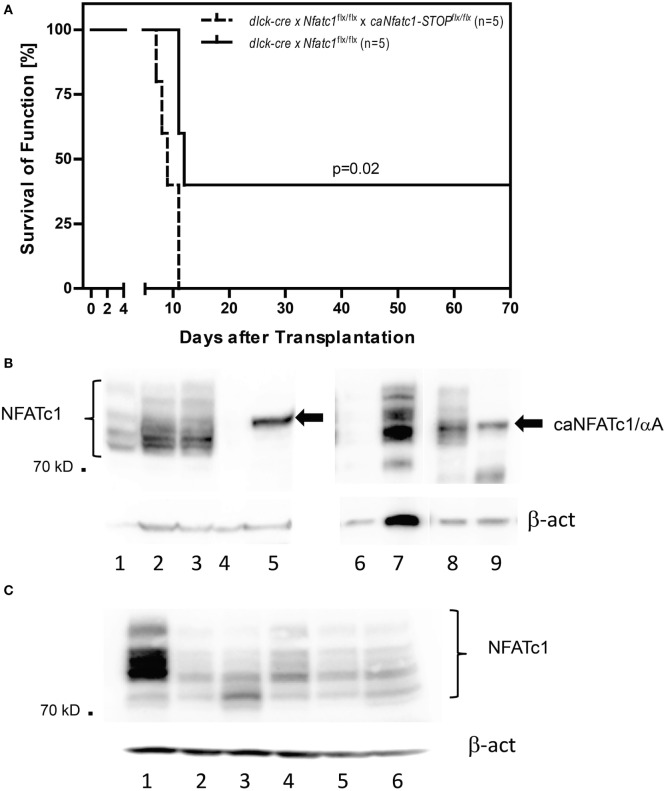
Expression of NFATc1/αA in peripheral T cells leads to rejection of allogeneic heterotopic heart transplants. **(A)** Allogenic heart grafts (of BALB/c mice) were transplanted into five *dlck-cre* x *Nfatc1^flx/flx^* mice, or five *dlck-cre x Nfatc1^flx/flx^ x caNfatc1-STOP^flx/flx^* mice expressing a constitutive active (ca) version of NFATc1/αA in peripheral T cells. **(B)** Immunoblots showing the expression of caNFATc1/αA in CD8^+^ T cells from two out of five *dlck-cre x Nfatc1^flx/flx^ x caNfatc1-STOP^flx/flx^* mice. In lanes 1 and 6, cytosolic protein, in lanes 2, 3, and 7 nuclear protein from wild-type (WT) CD8^+^ T cells stimulated by αCD3/CD28 antibodies (Abs) *in vitro* for 2 days was fractionated. In lanes 4 and 8, cytosolic protein, and in lanes 5 and 9 nuclear protein of CD8^+^ T cells activated for 2 days from *dlck-cre x Nfatc1^flx/flx^ x caNfatc1-STOP^flx/flx^* mice was fractionated. **(C)** Immunoblot showing NFATc1 expression in CD8^+^ T cells from five *dlck-cre x Nfatc1^flxl/flx^* mice. In lane 1, whole cell protein of WT CD8^+^ T cells stimulated by αCD3/CD28 Abs *in vitro* for 2 days was fractionated, in lanes 2–6, whole cell protein of activated CD8^+^ T cells from *dlck-cre x Nfatc1^flx/flx^* mice.

In order to demonstrate that—as we assumed—the induction of the short NFATc1 isoform NFATc1/αA, the most prominent NFATc1 protein in cytotoxic T cells (Figure 1B) (25), plays an important role in the rejection of allogeneic heart transplants, we used *caNfatc1-STOP^flx/flx^* mice as recipients. Those mice express a constitutive active (ca) version of NFATc1/αA (caNFATc1) from the murine Rosa26 locus upon cre-mediated removal of a “floxed” STOP sequence (21). However, to our surprise (over-) expressing caNFATc1/αA in thymocytes of *CD4-cre mice* was not tolerated since all T cells expressing solely caNFATc1/αA were eliminated during positive selection and did not appear in peripheral organs of mice (unpubl. data). Therefore, we crossed *caNfatc1-STOP^flx/flx^* mice with *dlck-cre* x *Nfatc1^flx/flx^* mice that express cre under the control of distal *lck* promoter and, thereby, caNFATc1/αA in peripheral T cells (but no endogenous NFATc1). In peripheral CD8^+^ T cells from all *dlck-cre x Nfatc1^flx/flx^x caNfatc1-STOP^flx/flx^* mice that we investigated, we observed caNFATc1/αA expression. In addition, each transplantation of allogeneic BALB/c hearts into *dlck-cre x Nfatc1^flx/flx^x caNfatc1-STOP^flx/flx^* mice led to acute rejection around day 9 (mean survival time, MST), comparable to MST observed in C57BL/6 WT mice expressing endogenous NFATc1 (Figures 2A,B).

Compared to the sustained survival of allogeneic transplanted hearts in the majority of *Nfatc1^flx/flx^x CD4-cre* recipient mice for 60 days and longer (Figure 1A), we detected a long-term survival (>60 days) of heart allografts in two out of five *dlck-cre x Nfatc1^flx/flx^* recipient mice (Figure 2A). In immunoblots using protein of activated CD8^+^ T cells from five *dlck-cre x Nfatc1^flx/flx^* mice, we detected numerous, albeit weak bands with the same or a similar size of the endogenous NFATc1 (Figure 2C). This might be due to an incomplete ablation of endogenous NFATc1 in splenic CD8^+^ T cells of *dlck-cre x Nfatc1^flx/flx^* mice. A deletion efficiency of approximately 80% has been reported for the *dlck-cre* line in CD8^+^ T cells, whereas a markedly higher efficiency rate was detected for the murine *CD4-cre* line (33).

### NFATc1 Ablation in T Cells Leads to a Decrease in Transcripts of Genes of T Cell Activation Signature in GICs

**Figure 3:**
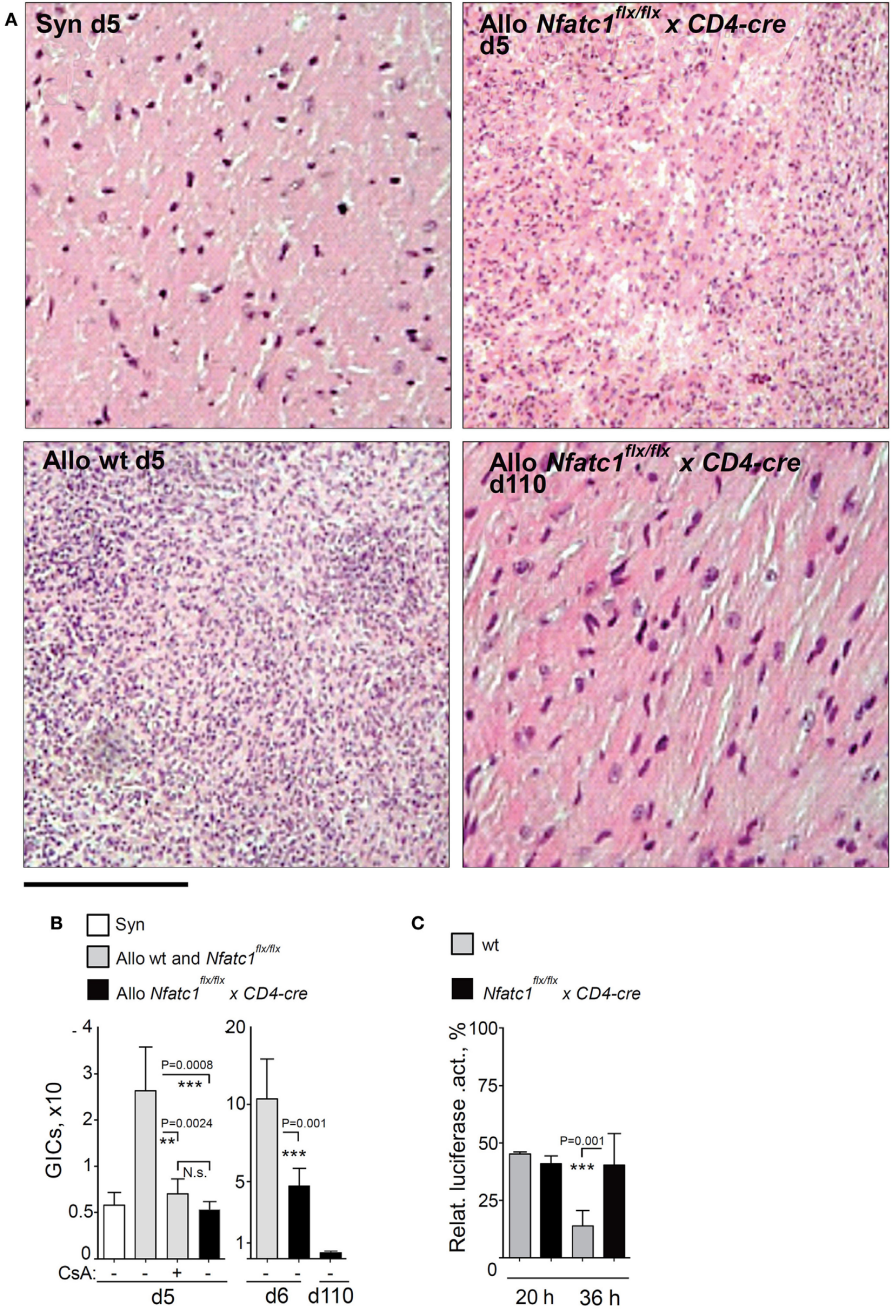
Graft-Infiltrating Cells (GICs) in transplanted hearts. **(A)** Representative hematoxylin–eosin stains of grafts from syngeneic and allogeneic wild-type (WT) recipients, and from *Nfatc1^flx/flx^ x CD4-cre* recipients during the acute rejection phase (d5), and from *Nfatc1^flx/flx^ x CD4-cre* recipients at d110. Bar, 300 µm. **(B)** Quantification of GICs in transplanted allogeneic hearts with (+) and without (−) cyclosporin A (CsA) treatment of animals. **(C)** Cytotoxicity of GICs. Luciferase assays were performed after cultivation of MOPC-*luciferase* target cells together with equal numbers of GICs isolated from WT or *Nfatc1^flx/flx^ xCD4-cre* recipient mice during the acute rejection phase (d6, effector/target ratio 4/1, followed by 20 or 36 h incubation).

In allogeneic BALB/c hearts, we observed a threefold to fivefold increase of GICs at days 5 and 6 during the acute rejection phase, compared to the number of GICs in transplanted syngeneic hearts (Figures 3A,B). However, approximately the same number of GICs we detected in allogeneic hearts transplanted into *Nfatc1^flx/flx^x CD4-cre* mice (Figure 3B), and in long-term accepted heart transplants (>100 days after transplantation) a further decrease in number of GICs was detected (Figure 3B). Compared to GICs isolated from rejected hearts, the cytotoxicity of GICs from accepted hearts of *Nfatc1^flx/flx^x CD4-cre* mice was significantly impaired, reaching approximately one-third of cytotoxicity of GICs from rejected hearts (Figure 3C).

**Figure 4:**
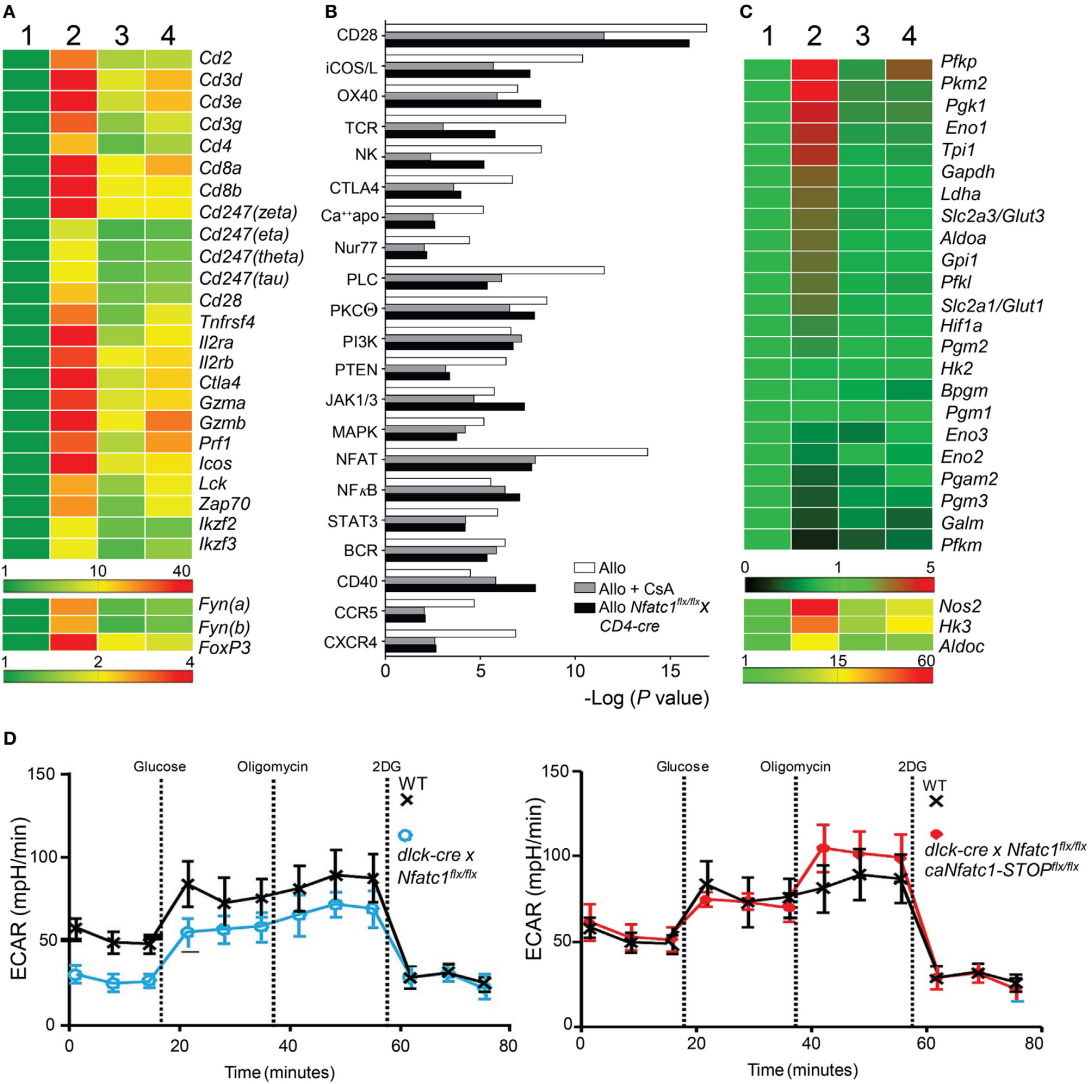
Cyclosporin A (CsA)-sensitive nuclear factors of activated T cell (NFAT) signaling is a major transcriptional pathway in Graft-Infiltrating Cells (GICs) from allogeneic heterotopic heart transplants. Syngeneic (C57BL/6, Syn) or fully allogeneic (BALB/c, Allo) heart grafts were transplanted into wild-type (WT) or NFATc1-deficient (*Nfatc1^flx/flx^xCD4-cre*) C57BL/6 recipients. A subgroup of WT recipients was treated daily with CsA (15 mg/kg, Allo CsA). GICs were purified during acute rejection phase (d5) and subjected to next-generation sequencing (NGS) assays. **(A)** RNA expression profiles (“heat map”) of T cell signature genes. GICs were purified from syngeneic recipients (lane 1), allogeneic recipients in the absence or presence of CsA (lanes 2 and 3, respectively), and from recipients bearing *Nfatc1*^−/−^ T cells (lane 4). Their RNAs were isolated and subjected to NGS. **(B)** Ingenious Pathway Analyses. Expression data were normalized to the transcriptome of GICs in syngeneic transplantations. **(C)** RNA expression of genes encoding glycolytic enzymes. **(D)** Extracellular flux analyses of activated CD8^+^ Ts from WT and *dlck-cre x Nfatc1^flx/flx^* mice (left), and from WT and *dlck-cre x Nfatc1^flx/flx^ x caNfatc1-STOP^flx/flx^* mice (right). Data of three independent assays are shown.

To define molecular targets of NFATc1 in GICs of transplanted hearts, we determined by next-generation sequencing (NGS) the transcriptomes of GICs isolated from syngeneic and allogeneic grafts on day 5 after transplantation, i.e., during the acute rejection phase of allogeneic grafts, and from allogeneic grafts of *Nfatc1^flx/flx^x CD4-cre* mice. Some of the B6 WT recipients of allogeneic grafts were treated with CsA (*n* = 5). In GICs from untreated allografts, the transcript levels of T cell signature genes (e.g., of *Cd3g,d,e, Cd4, Cd8a,b*, and *Il2ra,rb* genes) and of genes coding for signal molecules in T cells (of *Lck, Zap70*, and *Fyn* genes) were found to be increased, compared to GICs from syngeneic mice. In GICs from *Nfatc1^fl/fl^x CD4-cre* and CsA-treated WT recipients the transcript levels were lower, compared to GICs from untreated allogeneic recipients (Figure 4A). This is also true for the *Gzmb* and *Prf1* genes encoding the most prominent granzymes and perforins in CTLs.

The rejection of heart allografts is executed by various subsets of cells of adaptive and innate immune system. The population of GICs has been characterized by flow cytometry (34) but not at the transcriptome level. We hypothesized that the transcription of numerous genes that control signaling pathways for the activation, survival, and migration of GICs become activated in the majority of GICs, and suppressed by CsA. Analyses of signaling pathway activity (Figure 4B) revealed the expected activation and CsA-sensitivity of several T cell activation pathways, including the signatures of NK cell activity and activation of CD28 co-stimulatory pathway (35). Among them are the NF-κB and NFAT signatures that were found to be activated during the acute rejection phase. However, while NF-κB signaling remained unaffected, NFAT signaling in GICs was markedly suppressed by CsA treatment and in *Nfatc1^flx/flx^x CD4-cre* mice (Figure 4B). From these data, one may conclude that NFAT signaling is a major CsA-sensitive transcriptional pathway activated in GICs during acute rejection phase of fully allogeneic heart transplants.

Among the genes whose transcript levels increased upon transplantation and decreased by CsA treatment or NFATc1 ablation, there were numerous genes that encode enzymes of aerobic glycolysis (Figure 4C). This metabolic switch from OXPHOS to aerobic glycolysis is a typical sign of activated lymphocytes which have a high demand for energy and cellular building blocks, such as nucleotides and amino acids (36). To elucidate whether NFATc1 indeed plays a role in the control of aerobic glycolysis, we studied glycolysis by extracellular flux analysis in WT and *Nfatc1*^−/−^ CD8^+^ T cells from *dlck-cre x Nfatc1^flx/flx^* mice that we used in transplantation experiments (Figure 2A). As seen in Figure 4D, NFATc1 ablation in peripheral T cells by dlck-cre led to a marked decrease in ECAR which reflects the metabolic switch to glycolysis upon T cell activation. However, when—on the background of deficiency of endogenous NFATc1—the ca version of NFATc1/αA was expressed (Figure 4B), ectopic expression of NFATc1/αA exerted an overall increase in ECAR (Figure 4D), reflecting a stimulation of glycolysis and glycolytic capacity of CD8^+^ T cells by NFATc1/αA. These data suggest that NFATc1 supports the switch from OXPHOS to aerobic glycolysis during activation of peripheral CD8^+^ T cells. They are in line with the observation that inhibiting aerobic glycolysis in T cells suppressed the rejection of allogeneic heart transplants (37).

We were also interested in elucidating whether the transplantation of an allogeneic heart at d6, i.e., during the acute rejection phase, affected the peripheral T cell compartment in WT mice, in WT mice treated with CsA and in *Nfatc1^flx/flx^x CD4-cre* mice bearing NFATc1-deficient T cells. However, upon transplantation we detected only subtle differences in the composition of splenic T cells from these three types of mice (Figure S3 in Supplementary Material).

### NFATc1 Controls the Expression of *Cxcr3* Gene

**Figure 5:**
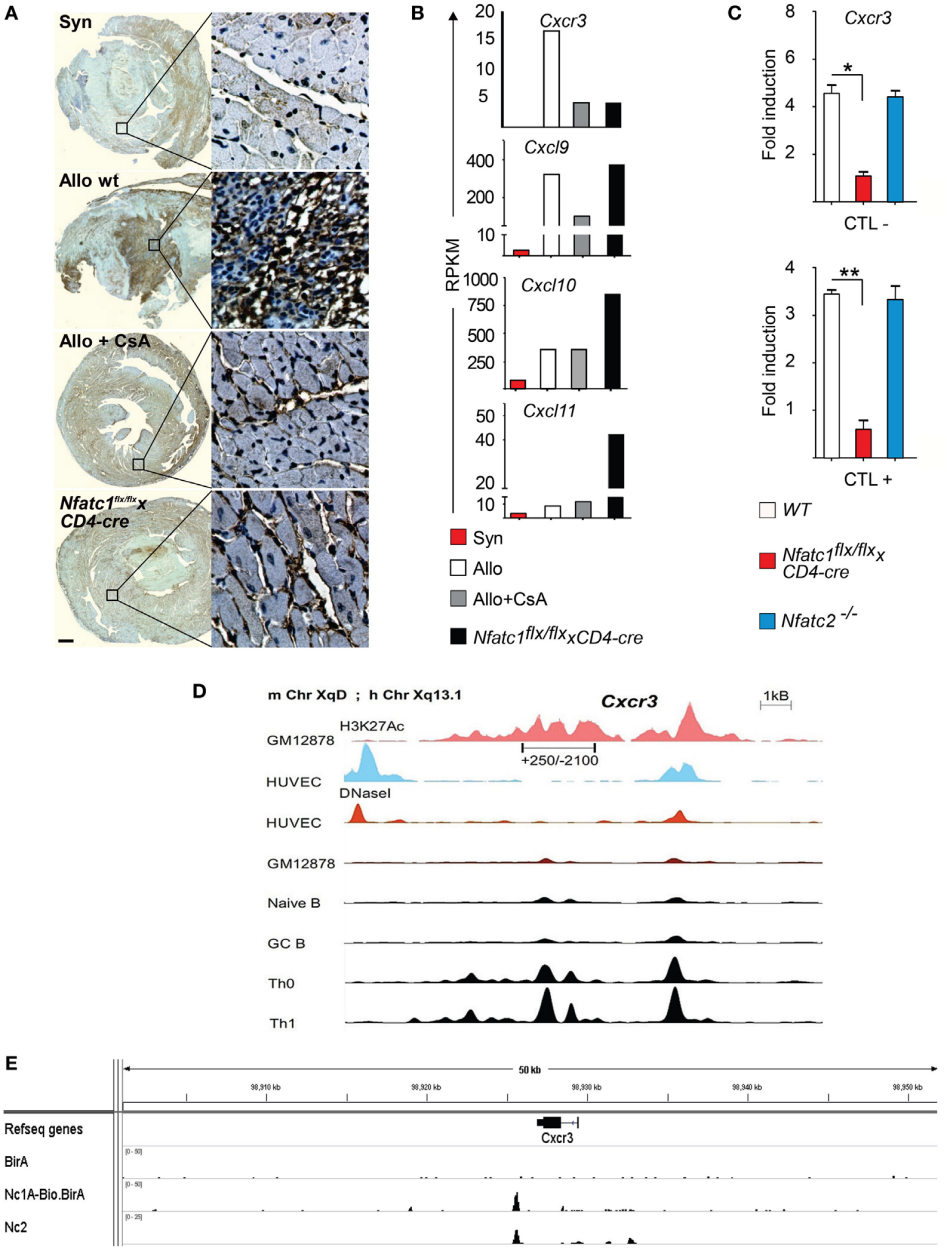
The *Cxcr3* gene is a direct NFATc1 target. **(A)** Representative immune histochemical stains of Cxcr3. Syngeneic (Syn) or allogeneic (Allo) heart grafts were transplanted into wild-type (WT) or cyclosporin A (CsA)-treated WT mice, or in recipient mice bearing NFATc1-deficient T cells (*Nfatc1^flx/flx^ x CD4-cre*). Grafts and graft-infiltrating cells (GICs) were analyzed during the acute rejection phase on day 5. Bar, 500 µm. **(B)** Next-generation sequencing assays. Normalized RNA expression of *Cxcr3* gene and of relevant ligand genes—*Cxcl9, Cxcl10*, and *Cxcl11—*in the transcriptome of GICs. RPKM, reads per kilobase per million reads. **(C)** Induction of *Cxcr3* transcripts in CTL cells derived from WT, *Nfatc1^flx/flx^ x CD4-cre* or *Nfatc2*^−/−^ mice. Real-time PCR assays of RNAs were normalized to *Actb* expression. **(D)** Genomic organization of the *Cxcr3* locus. The distribution of epigenetic modification histone mark H3K27Ac and the presence of DNaseI hypersensitive sites in non-expressing and cells expressing (as Th1 cells) is indicated. **(E)** Binding of NFATc1/A-Bio and NFATc2 (39) to the *Cxcr3* locus in CTLs. ChIP seq assays using a bacterial artificial chromosome transgenic mice expressing NFATc1/A-Bio protein (see Figure S4 in Supplementary Material).

The expression of chemokines and chemokine receptors plays a crucial role in the migration of peripheral T cells to inflamed organs. Cxcr3 expression was shown to determine the balance between effector and memory T cells (38) and to be required for acute allogeneic heart rejection (12). Histochemical stains of Cxcr3 expression revealed high levels of Cxcr3 in cells of allogeneic heart transplants whereas a strong decrease in Cxcr3 levels was observed in heart allografts of CsA-treated B6 WT or in *Nfatc1^flx/flx^x CD4-cre* mice (Figure 5A). In addition, the data of transcriptome assays showed a strong increase in *Cxcr3* transcripts in GICs isolated from allogeneic heart transplants and much less *Cxcr3* transcripts in GICs isolated from hearts transplanted into CsA-treated or *Nfatc1^flx/flx^x CD4-cre* mice (Figure 5B). These findings are supported by quantitative real-time polymerase chain reaction assays of RNAs from murine CTLs that show a marked decrease of *Cxcr3* transcripts in *Nfatc1*^−/−^ CTLs (Figure 5C).

In murine T cells, the *Cxcr3* locus is characterized by numerous DNase I hypersensitive chromatin sites in front of the *Cxcr3* gene, and extended H3K27 acetylation both in front and within the gene (Figure 5D). However, we were unable to trans-activate the immediate upstream promoter region (spanning the nucleotides from +250 to −2100) cloned in front of a luciferase reporter gene by NFATc1 (over-) expression in Jurkat T cells (data not shown). ChIP seq assays of CTLs that were generated *in vitro* from splenic CD8^+^ T cells of a novel tg mouse line expressing NFATc1/A-Bio protein (Figure S4 in Supplementary Material) (25) revealed strong NFATc1 binding downstream of the *Cxcr3* gene, approximately 1 kb 3′ from its poly A addition site (Figure 5E, below). These data suggest that NFATc1 plays a critical role in the expression of *Cxcr3* gene in T cells (Figure 5A). Moreover, the data suggest that in part the effect of NFATc1 on heart allograft rejection is mediated by Cxcr3 expression.

## Discussion

The findings of our study presented here showed that ablation of *Nfatc1* induction in T cells prevented the rejection of heterotropic heart transplants. In contrast, the expression of a ca version NFATc1/αA in T cells supported heart graft rejection indicating an important function for NFATc1/αA in the T cell-mediated immune reaction against allogeneic heart grafts.

The deficiency of NFATc1 in recipient mice resulted in an impaired infiltration of graft and decreased cytotoxicity of GICs. While during acute heart rejection GICs correspond to lymphocytes, monocytes, and neutrophils (40), organ graft rejection is a T cell-dependent process (41, 42) and a massive CD8^+^ T cell infiltration and a high ratio of CD8^+^/CD4^+^ cells was reported for heart allograft rejection in mice (43). The fate of organ transplants is determined to a large part by the number of effector T cells. Regulatory T cells (Tregs) that are known to control the activation and expansion of effector T cells can also affect the fate of a transplanted organ. In a MHC class II-mismatched cardiac allograft model, acute rejection of the donor heart grafts was inhibited by CD4^+^CD25^+^ Tregs that restricted the clonal expansion of alloreactive T cells (44). While we did not observe an effect of NFATs on the generation of thymus-derived naturally occurring regulatory T cells (nTreg), the generation of peripherally induced Treg by TGF-β was highly dependent on NFAT expression (15). However, neither inactivation of NFATs did impair the suppressive capacity of Tregs (15, 45), nor did activation of Tregs lead to any NFATc1/αA induction (46). These findings prompted us to conclude that Tregs play only a minor, if any role during induction of transplant tolerance in mice bearing NFATc1-deficient T cells.

One may hypothesize that due to the MHC I and II mismatches of allogeneic graft, NFATc1 expression is rapidly induced in T lymphocytes of peripheral lymphoid organs, in particular in spleen (47). This might lead to the fast re-stimulation of effector CTLs and their migration into the allograft in which CTLs destroy the graft (48, 49). Among the “best” target genes of NFATc1 that we determined by NGS and ChIP seq assays of murine CTLs generated *in vitro* we detected numerous lymphokine, chemokine, and chemokine receptor genes (25). We showed here that the expression of *Cxcr3* gene, which was shown previously to play an important role in acute allograft rejection (12) is controlled by NFATc1 in T cells. However, Cxcr3 is not the only molecule of chemokine network that affects heart transplants (50). Ablation of Ccr4, a further chemokine receptor, led to prolonged survival of allogeneic murine hearts in a chronic transplant model (51), and priming of T cells through the receptor of hepatocyte growth factor, c-Met, *in vitro* resulted in the generation of “T cell cardiotropism,” including Cxcr4 and Ccr4 expression (52). The priming through c-Met affected also the expression of Ccl3 and Ccl4, two ligands of chemokine receptor Ccr5 that are strongly induced by NFATc1 in murine CTLs (25). Among the direct targets immediately downstream of NFATc1 in T cells is the *Irf4* gene (25). The TF IRF4 has been shown recently to control the rejection of allogeneic murine hearts upon heterotopic transplantation (53).

In summary, our data show that ablation of *Nfatc1* induction in T cells is sufficient to prevent the rejection of allogeneic heart transplants, whereas (over-) expressing NFATc1/αA led to fulminant rejection. These findings favor NFATc1/αA as molecular target to prevent graft rejection upon heart transplantation.

## Ethics Statement

Animal experiments were performed according to project licenses (acceptance AKZ 55.2-2531.01-80/10 from 22.10.2010 and AKZ 55.2-2531.01-63/11), which are approved and controlled by the Regierung von Unterfranken, Würzburg.

## Author Contributions

JB, CO, US, SK-H, KhM, TP, KrM, RW, C-TG, IK, and NM performed experiments, ES and AA designed the study, performed experiments, analyzed data, and wrote the manuscript, along with CO.

## Conflict of Interest Statement

The authors declare that the research was conducted in the absence of any commercial or financial relationships that could be construed as a potential conflict of interest.

## Abbreviations

Ab, antibody; AICD, activation-induced cell death; BAC, bacterial artificial chromosome; CsA, cyclosporin A; CN, Calcineurin; ECAR, extra cellular acidification rate; FK506, tacrolimus; GICs, graft-infiltrating cells; NFATs, nuclear factors of activated T cells; NGS, next-generation sequencing; OXPHOS, oxidative phosphorylation; qRT-PCR, quantitative real-time polymerase chain reaction; T + I, 12-O-tetradecanoylphorbol-13-acetate (TPA) + Ionomycin; TF, transcription factor; tg, transgenic; WT, wild type.

## References

[1] Azzi JR Sayegh MH Mallat SG. Calcineurin inhibitors: 40 years later, can’t live without. J Immunol (2013) 191(12):5785–91. 10.4049/jimmunol.1390055 24319282

[2] Liu J Farmer JD Jr Lane WS Friedman J Weissman I Schreiber SL. Calcineurin is a common target of cyclophilin-cyclosporin A and FKBP-FK506 complexes. Cell (1991) 66(4):807–15. 10.1016/0092-8674(91)90124-H 1715244

[3] Aliabadi A Cochrane AB Zuckermann AO. Current strategies and future trends in immunosuppression after heart transplantation. Curr Opin Organ Transplant (2012) 17(5):540–5. 10.1097/MOT.0b013e328358000c 22941325

[4] Sieber M Baumgrass R. Novel inhibitors of the calcineurin/NFATc hub – alternatives to CsA and FK506? Cell Commun Signal (2009) 7:25. 10.1186/1478-811X-7-25 19860902 PMC2774854

[5] Palkowitsch L Marienfeld U Brunner C Eitelhuber A Krappmann D Marienfeld RB. The Ca2+-dependent phosphatase calcineurin controls the formation of the Carma1-Bcl10-Malt1 complex during T cell receptor-induced NF-kappaB activation. J Biol Chem (2011) 286(9):7522–34. 10.1074/jbc.M110.155895 21199863 PMC3045007

[6] Frischbutter S Gabriel C Bendfeldt H Radbruch A Baumgrass R Dephosphorylation of Bcl-10 by calcineurin is essential for canonical NF-kappaB activation in Th cells. Eur J Immunol (2011) 41(8):2349–57. 10.1002/eji.201041052 21674474

[7] Li H Rao A Hogan PG. Interaction of calcineurin with substrates and targeting proteins. Trends Cell Biol (2011) 21(2):91–103. 10.1016/j.tcb.2010.09.011 21115349 PMC3244350

[8] Aramburu J Yaffe MB Lopez-Rodriguez C Cantley LC Hogan PG Rao A. Affinity-driven peptide selection of an NFAT inhibitor more selective than cyclosporin A. Science (1999) 285(5436):2129–33. 10.1126/science.285.5436.2129 10497131

[9] Choi JM Sohn JH Park TY Park JW Lee SK. Cell permeable NFAT inhibitory peptide Sim-2-VIVIT inhibits T-cell activation and alleviates allergic airway inflammation and hyper-responsiveness. Immunol Lett (2012) 143(2):170–6. 10.1016/j.imlet.2012.01.016 22342853

[10] Serfling E Avots A Klein-Hessling S Rudolf R Vaeth M Berberich-Siebelt F NFATc1/alphaA: the other face of NFAT factors in lymphocytes. Cell Commun Signal (2012) 10(1):16 10.1186/1478-811X-10-16 22764736 PMC3464794

[11] Orosz CG Wakely E Bergese SD VanBuskirk AM Ferguson RM Mullet D Prevention of murine cardiac allograft rejection with gallium nitrate. Comparison with anti-CD4 monoclonal antibody. Transplantation (1996) 61(5):783–91. 10.1097/00007890-199603150-00019 8607184

[12] Hancock WW Lu B Gao W Csizmadia V Faia K King JA Requirement of the chemokine receptor CXCR3 for acute allograft rejection. J Exp Med (2000) 192(10):1515–20. 10.1084/jem.192.10.1515 11085753 PMC2193193

[13] Zhou P Hwang KW Palucki DA Guo Z Boothby M Newell KA Impaired NF-kappaB activation in T cells permits tolerance to primary heart allografts and to secondary donor skin grafts. Am J Transplant (2003) 3(2):139–47. 10.1034/j.1600-6143.2003.00033.x 12603209

[14] Muhammad K Alrefai H Marienfeld R Pham DA Murti K Patra AK NF-kappaB factors control the induction of NFATc1 in B lymphocytes. Eur J Immunol (2014) 44(11):3392–402. 10.1002/eji.201444756 25179582

[15] Vaeth M Schliesser U Muller G Reissig S Satoh K Tuettenberg A Dependence on nuclear factor of activated T-cells (NFAT) levels discriminates conventional T cells from Foxp3+ regulatory T cells. Proc Natl Acad Sci U S A (2012) 109(40):16258–63. 10.1073/pnas.1203870109 22991461 PMC3479579

[16] Oh-Hora M Komatsu N Pishyareh M Feske S Hori S Taniguchi M Agonist-selected T cell development requires strong T cell receptor signaling and store-operated calcium entry. Immunity (2013) 38(5):881–95. 10.1016/j.immuni.2013.02.008 23499491 PMC3669219

[17] Aliprantis AO Ueki Y Sulyanto R Park A Sigrist KS Sharma SM NFATc1 in mice represses osteoprotegerin during osteoclastogenesis and dissociates systemic osteopenia from inflammation in cherubism. J Clin Invest (2008) 118(11):3775–89. 10.1172/JCI35711 18846253 PMC2564610

[18] Hobeika E Thiemann S Storch B Jumaa H Nielsen PJ Pelanda R Testing gene function early in the B cell lineage in mb1-cre mice. Proc Natl Acad Sci U S A (2006) 103(37):13789–94. 10.1073/pnas.0605944103 16940357 PMC1564216

[19] Hodge MR Ranger AM Charles de la Brousse F Hoey T Grusby MJ Glimcher LH. Hyperproliferation and dysregulation of IL-4 expression in NF-ATp-deficient mice. Immunity (1996) 4(4):397–405. 10.1016/S1074-7613(00)80253-8 8612134

[20] Lee PP Fitzpatrick DR Beard C Jessup HK Lehar S Makar KW A critical role for Dnmt1 and DNA methylation in T cell development, function, and survival. Immunity (2001) 15(5):763–74. 10.1016/S1074-7613(01)00227-8 11728338

[21] Baumgart S Chen NM Siveke JT Konig A Zhang JS Singh SK Inflammation-induced NFATc1-STAT3 transcription complex promotes pancreatic cancer initiation by KrasG12D. Cancer Discov (2014) 4(6):688–701. 10.1158/2159-8290.CD-13-0593 24694735 PMC4069603

[22] Zhang DJ Wang Q Wei J Baimukanova G Buchholz F Stewart AF Selective expression of the Cre recombinase in late-stage thymocytes using the distal promoter of the Lck gene. J Immunol (2005) 174(11):6725–31. 10.4049/jimmunol.174.11.6725 15905512

[23] Busch R Murti K Liu J Patra AK Muhammad K Knobeloch KP NFATc1 releases BCL6-dependent repression of CCR2 agonist expression in peritoneal macrophages from *Saccharomyces cerevisiae* infected mice. Eur J Immunol (2016) 46(3):634–46. 10.1002/eji.201545925 26631626

[24] de Boer J Williams A Skavdis G Harker N Coles M Tolaini M Transgenic mice with hematopoietic and lymphoid specific expression of Cre. Eur J Immunol (2003) 33(2):314–25. 10.1002/immu.200310005 12548562

[25] Klein-Hessling S Muhammad K Klein M Pusch T Rudolf R Floter J NFATc1 controls the cytotoxicity of CD8(+) T cells. Nat Commun (2017) 8(1):511. 10.1038/s41467-017-00612-6 28894104 PMC5593830

[26] Sitaru AG Timmermann W Ulrichs K Otto C. Hierarchical immunogenicity of donor MHC class I peptides in allotransplantation. Hum Immunol (2002) 63(10):871–9. 10.1016/S0198-8859(02)00452-4 12368039

[27] Alrefai H Muhammad K Rudolf R Pham DA Klein-Hessling S Patra AK NFATc1 supports imiquimod-induced skin inflammation by suppressing IL-10 synthesis in B cells. Nat Commun (2016) 7:11724. 10.1038/ncomms11724 27222343 PMC4894959

[28] Ebert A McManus S Tagoh H Medvedovic J Salvagiotto G Novatchkova M The distal V(H) gene cluster of the Igh locus contains distinct regulatory elements with Pax5 transcription factor-dependent activity in pro-B cells. Immunity (2011) 34(2):175–87. 10.1016/j.immuni.2011.02.005 21349430

[29] Robinson JT Thorvaldsdottir H Winckler W Guttman M Lander ES Getz G Integrative genomics viewer. Nat Biotechnol (2011) 29(1):24–6. 10.1038/nbt.1754 21221095 PMC3346182

[30] Ueno T Yamada A Ito T Yeung MY Gorbatov R Shimizu T Role of nuclear factor of activated T cell (NFAT) transcription factors in skin and vascularized cardiac allograft rejection. Transplantation (2011) 92(5):e26–7. 10.1097/TP.0b013e318228061c 21866037

[31] Serfling E Klein-Hessling S Palmetshofer A Bopp T Stassen M Schmitt E. NFAT transcription factors in control of peripheral T cell tolerance. Eur J Immunol (2006) 36(11):2837–43. 10.1002/eji.200536618 17039563

[32] Klein-Hessling S Rudolf R Muhammad K Knobeloch KP Maqbool MA Cauchy P A threshold level of NFATc1 activity facilitates thymocyte differentiation and opposes notch-driven leukaemia development. Nat Commun (2016) 7:11841. 10.1038/ncomms11841 27312418 PMC4915031

[33] Sharma S Zhu J. Immunologic applications of conditional gene modification technology in the mouse. Curr Protoc Immunol (2014) 105:10.34.1–13. 10.1002/0471142735.im1034s105 24700321 PMC4100247

[34] Stinn JL Taylor MK Becker G Nagano H Hasegawa S Furakawa Y Interferon-gamma-secreting T-cell populations in rejecting murine cardiac allografts: assessment by flow cytometry. Am J Pathol (1998) 153(5):1383–92. 10.1016/S0002-9440(10)65725-2 9811329 PMC1853393

[35] Maier S Tertilt C Chambron N Gerauer K Huser N Heidecke CD Inhibition of natural killer cells results in acceptance of cardiac allografts in CD28-/- mice. Nat Med (2001) 7(5):557–62. 10.1038/87880 11329056

[36] Finlay D Cantrell DA. Metabolism, migration and memory in cytotoxic T cells. Nat Rev Immunol (2011) 11(2):109–17. 10.1038/nri2888 21233853 PMC3521506

[37] Lee CF Lo YC Cheng CH Furtmuller GJ Oh B Andrade-Oliveira V Preventing allograft rejection by targeting immune metabolism. Cell Rep (2015) 13(4):760–70. 10.1016/j.celrep.2015.09.036 26489460 PMC4626381

[38] Hu JK Kagari T Clingan JM Matloubian M. Expression of chemokine receptor CXCR3 on T cells affects the balance between effector and memory CD8 T-cell generation. Proc Natl Acad Sci U S A (2011) 108(21):E118–27. 10.1073/pnas.1101881108 21518913 PMC3102421

[39] Martinez GJ Pereira RM Aijo T Kim EY Marangoni F Pipkin ME The transcription factor NFAT promotes exhaustion of activated CD8(+) T cells. Immunity (2015) 42(2):265–78. 10.1016/j.immuni.2015.01.006 25680272 PMC4346317

[40] Reichel CA Khandoga A Anders HJ Schlondorff D Luckow B Krombach F. Chemokine receptors Ccr1, Ccr2, and Ccr5 mediate neutrophil migration to postischemic tissue. J Leukoc Biol (2006) 79(1):114–22. 10.1189/jlb.0605337 16275892

[41] Whitby EH Sparshott SM Bell EB. Allograft rejection in athymic nude rats by transferred T-cell subsets. I. The response of naive CD4+ and CD8+ thoracic duct lymphocytes to complete allogeneic incompatibilities. Immunology (1990) 69(1):78–84. 2138126 PMC1385723

[42] Jones ND Van Maurik A Hara M Gilot BJ Morris PJ Wood KJ. T-cell activation, proliferation, and memory after cardiac transplantation in vivo. Ann Surg (1999) 229(4):570–8. 10.1097/00000658-199904000-00018 10203092 PMC1191745

[43] Szot GL Zhou P Rulifson I Wang J Guo Z Kim O Different mechanisms of cardiac allograft rejection in wildtype and CD28-deficient mice. Am J Transplant (2001) 1(1):38–46. 10.1034/j.1600-6143.2001.010108.x 12095035

[44] Schenk S Kish DD He C El-Sawy T Chiffoleau E Chen C Alloreactive T cell responses and acute rejection of single class II MHC-disparate heart allografts are under strict regulation by CD4+ CD25+ T cells. J Immunol (2005) 174(6):3741–8. 10.4049/jimmunol.174.8.5135-a 15749914

[45] Bopp T Palmetshofer A Serfling E Heib V Schmitt S Richter C NFATc2 and NFATc3 transcription factors play a crucial role in suppression of CD4+ T lymphocytes by CD4+ CD25+ regulatory T cells. J Exp Med (2005) 201(2):181–7. 10.1084/jem.20041538 15657288 PMC2212786

[46] Vaeth M Gogishvili T Bopp T Klein M Berberich-Siebelt F Gattenloehner S Regulatory T cells facilitate the nuclear accumulation of inducible cAMP early repressor (ICER) and suppress nuclear factor of activated T cell c1 (NFATc1). Proc Natl Acad Sci U S A (2011) 108(6):2480–5. 10.1073/pnas.1009463108 21262800 PMC3038697

[47] Larsen CP Morris PJ Austyn JM. Migration of dendritic leukocytes from cardiac allografts into host spleens. A novel pathway for initiation of rejection. J Exp Med (1990) 171(1):307–14. 10.1084/jem.171.1.307 2404081 PMC2187651

[48] Gilot BJ Hara M Jones ND van Maurik A Niimi M Hadjianastassiou V Visualization of the in vivo generation of donor antigen-specific effector CD8+ T cells during mouse cardiac allograft rejection: in vivo effector CD8+ T cell generation during allograft rejection. Transplantation (2000) 69(4):639–48. 10.1097/00007890-200002270-00028 10708123

[49] Delfs MW Furukawa Y Mitchell RN Lichtman AH. CD8+ T cell subsets TC1 and TC2 cause different histopathologic forms of murine cardiac allograft rejection. Transplantation (2001) 71(5):606–10. 10.1097/00007890-200103150-00005 11292288

[50] O’Boyle G Ali S Kirby JA. Chemokines in transplantation: what can atypical receptors teach us about anti-inflammatory therapy? Transplant Rev (Orlando) (2011) 25(4):136–44. 10.1016/j.trre.2010.10.005 21514134

[51] Huser N Tertilt C Gerauer K Maier S Traeger T Assfalg V CCR4-deficient mice show prolonged graft survival in a chronic cardiac transplant rejection model. Eur J Immunol (2005) 35(1):128–38. 10.1002/eji.200324745 15593118

[52] Komarowska I Coe D Wang G Haas R Mauro C Kishore M Hepatocyte growth factor receptor c-Met instructs T cell cardiotropism and promotes T cell migration to the heart via autocrine chemokine release. Immunity (2015) 42(6):1087–99. 10.1016/j.immuni.2015.05.014 26070483 PMC4510150

[53] Wu J Zhang H Shi X Xiao X Fan Y Minze LJ Ablation of transcription factor IRF4 promotes transplant acceptance by driving allogenic CD4(+) T cell dysfunction. Immunity (2017) 47(6):1114–1128.e6. 10.1016/j.immuni.2017.11.003 29221730 PMC5759774

