# Supplementary material for: The Transcription Factor NFATc1 Supports the Rejection of Heterotopic Heart Allografts
Source: Front Immunol. 2018 Jun 12;9:1338. doi: 10.3389/fimmu.2018.01338 (PMC6005848; doi:10.3389/fimmu.2018.01338)
Supplement: Supplementary file 1 [file Presentation_1.PDF]

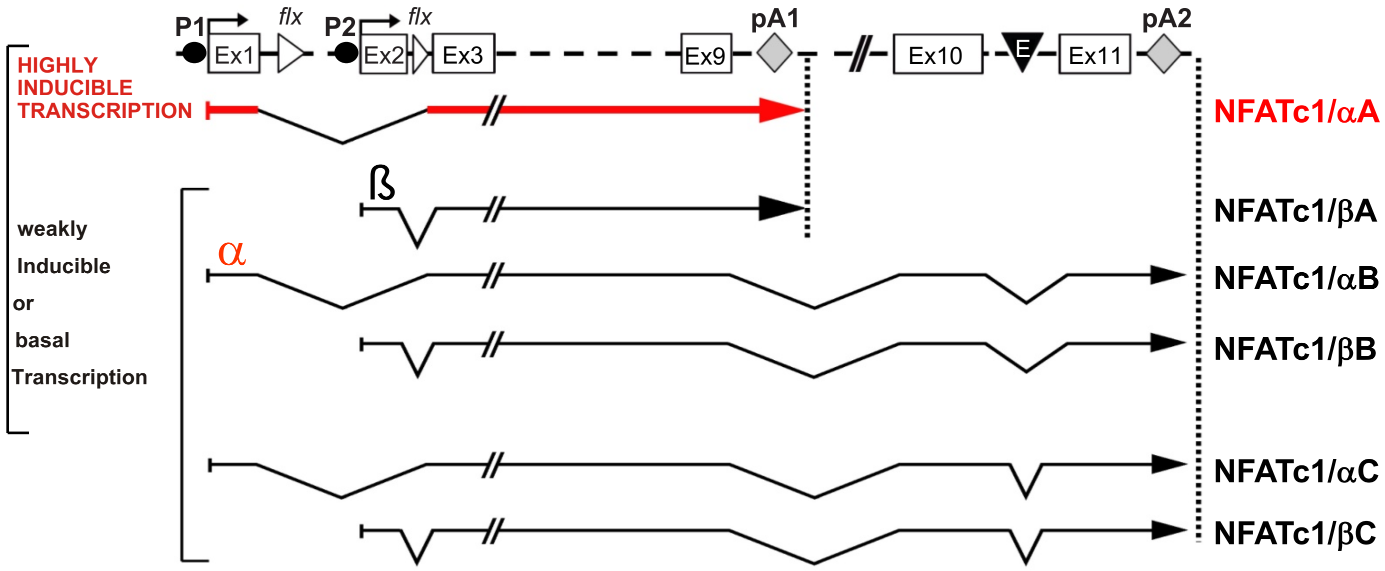

Supplementary Figure 1. Scheme of the murine *Nfatc1* gene and its expression in lymphoid cells (adapted from (32)). The positions of the promoters P1 and P2, of the polyA addition sites pA1 and pA2, and of the remote intronic enhancer E are indicated. The *flx* sites used for the deletion of P2 promoter and exon 2 in *Nfatc2P2Δ* mice (33) are shown as triangles. Below, the generation of six alternative *Nfatc1* isoforms is shown. The α- and β-peptides are generated from P1- or P2-directed exon 1 and exon 2 transcripts, respectively. They correspond to the N-terminal peptides of NFATc1/α and β-isoforms, respectively.

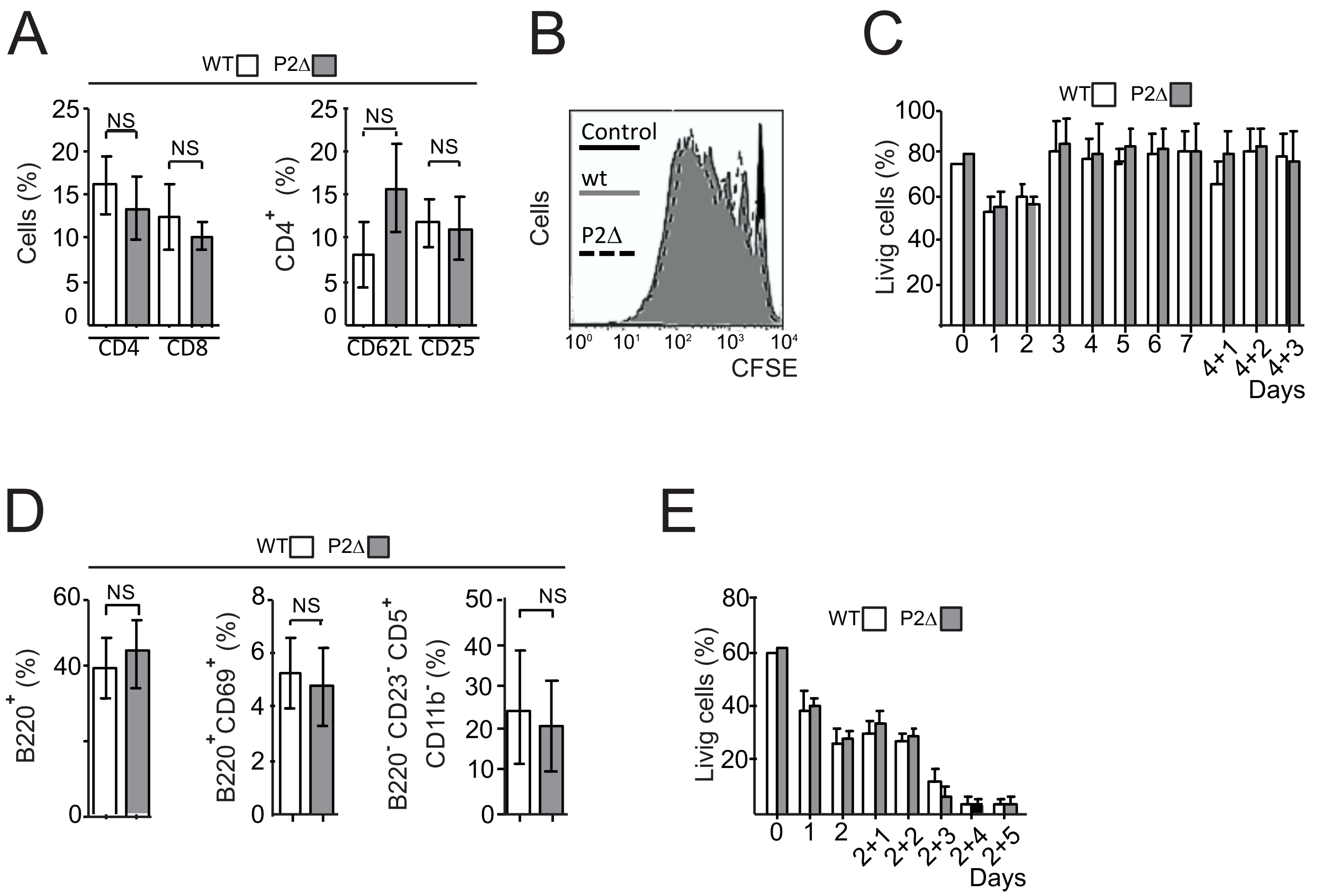

Supplementary Figure 2. Normal lymphoid compartments in *Nfatc1P2Δ* mice. (A) Left, percentage of CD4$^+$ and CD8$^+$T cells in spleen, and expression of the activation markers CD62L and CD25 on splenic CD4$^+$ T cells (right). (B) CD4$^+$T cells were isolated from spleens of WT and *Nfatc1P2Δ* littermate mice, labelled with CFSE and stimulated with Abs directed against CD3 and CD28 for 72 h. The proliferation of T cells was analysed by flow cytometry. (C) CD4$^+$T cells were stimulated with Abs against CD3 and CD28 and re-stimulated on d4 for 3 additional days. AICD was analysed by flow cytometry after annexin V and propidium iodine staining. (D) Percentage of B220$^+$ B-cells in spleen (left), expression of CD69 on splenic B-cells (middle) and percentage of peritoneal B1a cell population (right). (E) AICD of naïve B220$^+$ splenic B-cells stimulated with Ab directed against IgM and re-stimulated at d2 for 5 additional days.

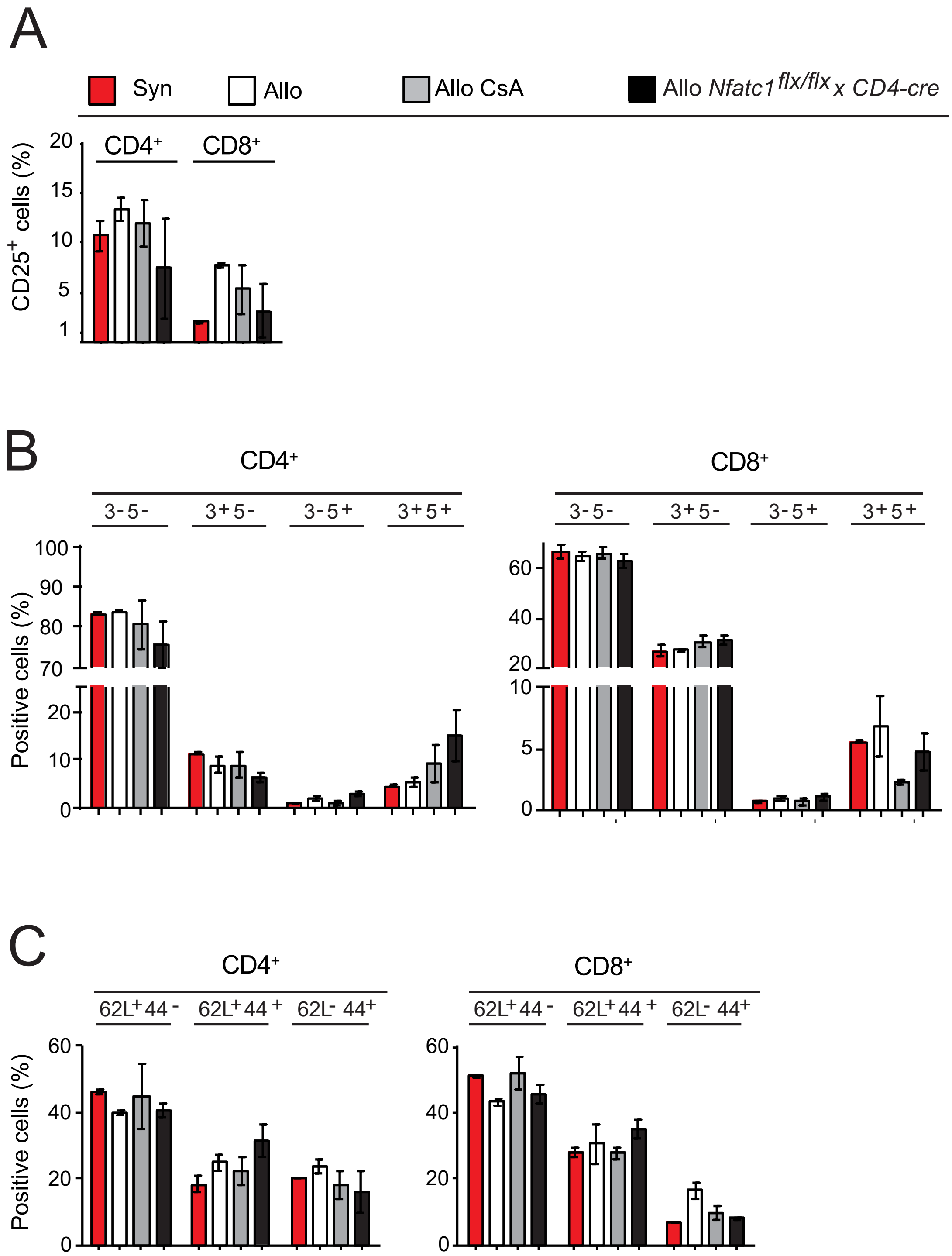

Supplementary Figure 3. NFATc1 deficiency and CsA-treatment affect peripheral T cell responses in a similar way. Flow cytometry of splenic T cells during the acute rejection phase (d6) after indicated heterotopic heart transplantations. (A) Expression of CD25 activation marker on $CD4^+$ and $CD8^+$ T cells. (B) Analyses of Cxcr3 (3) and Ccr5 (5) expression. (C) Analyses of naïve T ($CD62^+CD44^-$), T_{CM} ($CD62L^+CD44^+$) and T_{EM} ($CD62L^-CD44^+$) cell populations.

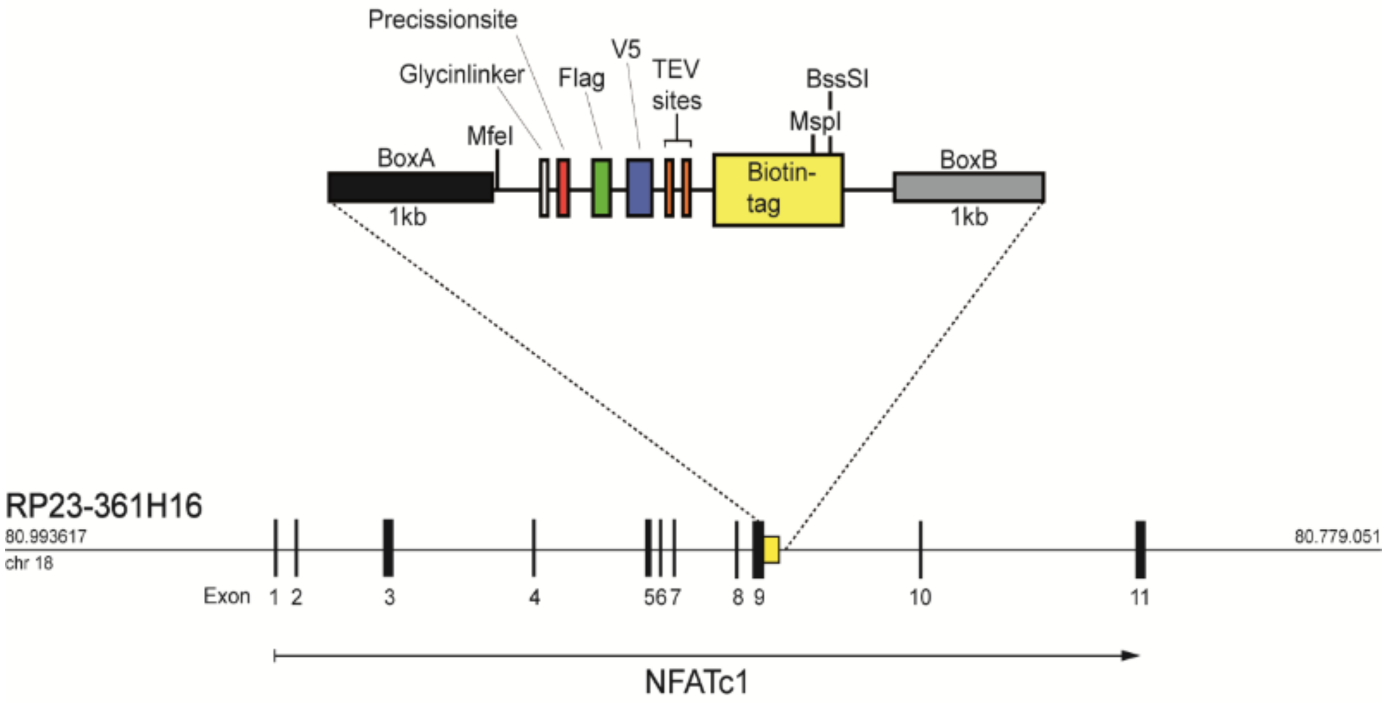

Supplementary Figure 4. Scheme of the *Nfatc1/A-Bio* BAC construct that was used to create transgenic *Nfatc1/A-Bio* mice. Above, the Biotin-tag is shown that was inserted behind exon 9 of the *Nfatc1* gene into the BAC RP23-361H16 containing the murine *Nfatc1* gene (mm9 chr.18, 80,779,051–80,993,617, 214 kb) (for details see (25)).
